# A 5-Year Mortality Prediction Model for Prostate Cancer Patients Based on the Korean Nationwide Health Insurance Claims Database

**DOI:** 10.3390/jpm14101058

**Published:** 2024-10-13

**Authors:** Joungyoun Kim, Yong-Hoon Kim, Yong-June Kim, Hee-Taik Kang

**Affiliations:** 1 Department of Artificial Intelligence, University of Seoul, Seoul 02540, Republic of Korea; joungyoun@uos.ac.kr; 2 Department of Biostatistics and Computing, Yonsei University Graduate School, Seoul 03722, Republic of Korea; asyhkim12@yuhs.ac; 3 Department of Urology, Chungbuk National University Hospital, Cheongju 28644, Republic of Korea; 4 Department of Urology, College of Medicine, Chungbuk National University, Cheongju 28644, Republic of Korea; 5 Department of Family Medicine, Severance Hospital, Yonsei University College of Medicine, Seoul 03722, Republic of Korea

**Keywords:** prostate neoplasms, survival, survival rate, probability, area under curve

## Abstract

Background: Prostate cancer is the fourth most common cancer and eighth leading cause of cancer-related mortality worldwide. Its incidence is increasing in South Korea. This study aimed to investigate a predictive model for the 5-year survival probability of prostate cancer patients in a Korean primary care setting. Method: This retrospective study used data from the nationwide insurance claims database. The main outcome was survival probability 5 years after the initial diagnosis of prostate cancer. Potential confounding factors such as age, body mass index (BMI), blood pressure, laboratory results, lifestyle behaviors, household income, and comorbidity index were considered. These variables were available in the national health check-up information. A Cox proportional hazards regression model was used to develop the predictive model. The predictive performance was calculated based on the mean area under the receiver operating characteristic curve (AUC) after 10-fold cross-validation. Results: The mean 5-year survival probability was 82.0%. Age, fasting glucose and gamma-glutamyl transferase levels, current smoking, and multiple comorbidities were positively associated with mortality, whereas BMI, alkaline phosphatase levels, total cholesterol levels, alcohol intake, physical activity, and household income were inversely associated with mortality. The mean AUC after 10-fold cross-validation was 0.71. Conclusions: The 5-year survival probability model showed a moderately good predictive performance. This may be useful in predicting the survival probability of prostate cancer patients in primary care settings. When interpreting these results, potential limitations, such as selection or healthy user biases, should be considered.

## 1. Introduction

Cancer statistics in South Korea (hereinafter, Korea) reported that malignant neoplasms are the leading cause of death in Korea, even though mortality rates due to cancers have declined since 2002 [1]. Globally, the disease burden of malignant neoplasms has increased [2]. Prostate cancer is the fourth most common cancer and eighth leading cause of cancer-related mortality worldwide [3]. In 2021, the age-standardized prostate cancer incidence and mortality per 100,000 individuals in Korea were 34.5 and 4.0, respectively [4]. Age-standardized prostate cancer incidence and mortality ranked fourth and seventh in 2021 [4]. The incidence and mortality of prostate cancer has steadily increased since 1999. The National Cancer Screening Programs provided by the National Health Insurance Service (NHIS) include screening for five types of cancer (stomach, colorectum, liver, breast, and uterine cervix) but do not include prostate cancer screening [5]. The NHIS does not provide routine screening for prostate cancer because it is unclear whether prostate-specific antigen (PSA)-based screening can reduce prostate cancer-related or overall mortality and be cost-effective [6,7].

The 5-year relative survival rate of prostate cancer patients in Korea increased from 59.1% in 1993–1995 to 96.0% in 2017–2021 [4]. The mortality of prostate cancer is significantly affected by pathological stage and Gleason score [8]. In addition, age at diagnosis, comorbidities, body mass index (BMI), economic status, and lifestyle behaviors such as cigarette smoking, alcohol intake, and physical activity are associated with the deaths of patients with various types of cancer including prostate cancer [9,10,11,12,13]. If the survival probability of prostate cancer patients can be estimated based on health check-up information and personal health records, primary care doctors and prostate cancer specialists could provide them with appropriate health care and resources according to their life expectancy.

Therefore, this study aimed to develop a survival prediction model for patients with prostate cancer using national health check-up data that are easily available in outpatient clinics. Thus, this model may be useful for predicting the 5-year survival probability in primary care settings, where information regarding the pathological stage, Gleason score, or anti-cancer treatment details is difficult to obtain.

## 2. Materials and Methods

### 2.1. Data Source

This retrospective cohort study was designed using the NHIS Health Screening cohort from the Korean National Health Information Database (NHID). Almost all Koreans are subscribed to the NHIS (approximately 97%), with the exception of Medicaid beneficiaries (approximately 3%) who have very low household incomes. The NHIS collects insurance information (type and premium based on annual household income); information regarding medical utilization, including hospital visits, medical records (such as prescriptions, treatments, and diagnostic codes), and medical expenses; death information (date and cause of death) from death certificates; and data from health check-ups. The NHIS provides adults over 40 years of age with a biennial national health screening program during which the following details are collected: anthropometric data (height and body weight); blood pressure; lifestyle behaviors, such as cigarette smoking, alcohol intake, and physical activity; personal medical history; and laboratory test results, including fasting glucose, alanine aminotransferase (ALT), gamma-glutamyl transferase (GGT), and total cholesterol concentrations. The NHIS collects and provides these data to researchers for research purposes only. The last date that data were accessed for this study was 1 April 2024.

The institutional review board of Severance Hospital, Yonsei University Health System approved this study on 28 November 2023 (IRB No. 4-2023-1318). This study complied with the tenets of the 1964 Helsinki Declaration.

### 2.2. Study Population and Definition of Malignant Neoplasms

**Figure 1:**
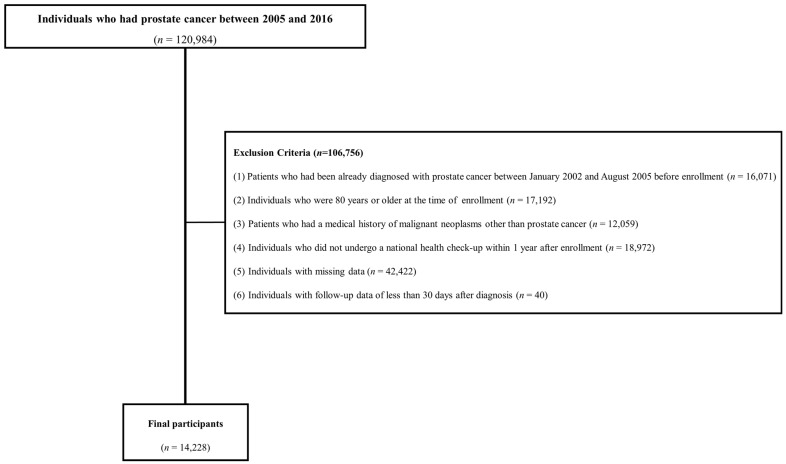
Flowchart of study population.

Figure 1 shows the flowchart of participants according to the inclusion and exclusion criteria. This cohort included patients with a prostate cancer diagnosis between 2002 and 2021. To enroll patients newly diagnosed with prostate cancer and rule out false positives for prostate cancer, those diagnosed before September 2005 when the special code system began were excluded from the study. Initially, 120,984 individuals diagnosed with prostate cancer between 2005 and 2016 were included in the study. The following exclusion criteria were applied: (1) patients who had been already diagnosed with prostate cancer between January 2002 and August 2005 before enrollment (*n* = 16,071); (2) individuals aged ≥ 80 years at the time of enrollment (*n* = 17,192); (3) patients with a history of malignant neoplasms other than prostate cancer (*n* = 12,059); (4) individuals who did not undergo a national health check-up within one year of being newly diagnosed with prostate cancer (*n* = 18,972); (5) individuals with missing data (*n* = 42,422); and (6) individuals with follow-up data of less than 30 days after diagnosis (*n* = 40). In total, 14,228 men were included in the final analysis.

Patients with malignant neoplasms were defined using the special codes V193 or V194 in their medical records. Special codes are normally assigned for severe refractory diseases, such as cardiovascular diseases or end-stage kidney diseases, and rare diseases, such as congenital anomalies or severe metabolic diseases, in addition to malignant neoplasms [14]. Patients with special diseases are more strictly monitored than patients with more common or less refractory diseases because the NHIS covers more medical expenditure for these special diseases. Among the special codes, V193 and V194 indicate malignant neoplasms. The types of malignant neoplasms are defined according to the combination of the V193 or V194 codes and the main diagnostic codes, which are based on the International Classification of Diseases (ICD)-10th edition codes for cancer (C00–C99). Prostate cancer is defined as both the C61 and the V193 or V194. This strict definition minimizes selection bias against false positives or negatives.

### 2.3. Prediction Variables

Variables obtained from health check-ups and personal medical records were selected as potential predictors of mortality in prostate cancer patients. Age, BMI, socioeconomic status, and lifestyle factors such as smoking, alcohol intake, and physical activity are associated with death in patients with various types of cancer, including prostate cancer [9,10,11,12,13]. Patients with prostate cancer often die from non-prostate cancer-related causes, such as cardiovascular disease [15]. Thus, the risk factors for cardiovascular diseases should be considered when developing a prognostic model. Age, BMI, blood pressure, fasting glucose, ALT, GGT, total cholesterol, and lifestyle factors were controlled in the present study [16,17]. These variables are available in the health check-up database from the NHID and commonly measured in primary care settings.

Continuous variables included age, BMI, systolic blood pressure (SBP), and laboratory test results (fasting glucose, ALT, GGT, and total cholesterol levels). Categorical variables included health behaviors (cigarette smoking, alcohol intake, and physical activity), economic status (based on annual household income), and the Charlson Comorbidity Index (CCI) score. Patients were categorized as never (those who had never smoked cigarettes), former (those who had smoked cigarettes in the past, but not at the time of enrollment), or current smokers (those who currently smoked cigarettes at the time of enrollment). Patients were classified as rare, moderate, or heavy drinkers. Rare drinkers were individuals who consumed alcohol ≤ three times per month, moderate drinkers were those who consumed alcohol one to four times per week, and heavy drinkers were those who consumed alcohol five or more times per week. Physical activity was categorized into low, moderate, and high levels. A low level was defined as engaging in physical activity < two times per week; moderate, three or four times per week; and high, five or more times per week. In terms of annual household income, patients were categorized into five groups according to insurance premiums.

The CCI is used to categorize patients according to their comorbidities based on the ICD codes recorded in administrative data [18]. Each comorbidity has weighted values from 1 to 6 based on the adjusted risk of death or associated resource use. The sum of all the weights provides a single comorbidity score for each patient. A score of zero means that no serious comorbidities are detected. A higher CCI score indicates a greater likelihood that a condition will result in death or higher medical resource use [19]. In this study, patients were categorized into four groups based on the CCI score (0, 1, 2, and 3 or more).

### 2.4. Statistical Analysis

Continuous variables (age, BMI, SBP, fasting glucose, ALT, GGT, and total cholesterol levels) are presented as the mean ± standard deviation. Categorical variables (cigarette smoking, alcohol intake, physical activity, annual household income, and CCI score) are expressed as the number of patients (percentage).

A Cox proportional hazards regression model was used to develop a prediction model for the 5-year mortality of prostate cancer patients [20,21]. Patients were censored on the date of their last clinic visit if they did not die during the study period. Potential risk factors, such as age, BMI, SBP, laboratory results (fasting glucose, total cholesterol, ALT, and GGT levels), lifestyle behaviors (smoking, alcohol intake, and physical activity), economic status, and CCI score, were adjusted in the Cox proportional hazards regression model. Except for the CCI score, these variables were collected from the National Health Check-Up Program. The proportional hazards assumption was verified by investigating the Schoenfeld residuals. Additionally, the logarithm of the cumulative hazard function was estimated based on Kaplan–Meier curves.

The 5-year mortality probability for a patient with prostate cancer can be estimated using the following equation:

$$P\left(survival\;time\leq t\right)=1-S_{0}(t){exp}^{\beta_{1}\left(x_{1}-M_{1}\right)+\beta_{2}\left(x_{2}-M_{2}\right)+\cdots +\beta_{p}(x_{p}-M_{p})}$$

where *S*_0_(*t*) is the survival rate at the mean value of the risk factor at time *t*. In this study, the 5-year survival rate, *S*_0_(5), was estimated to be 0.820. *β_j_* and *M_j_* (*j* = 1, 2, … *p* = 12) are the regression coefficients in the Cox proportional hazards model and the sample mean of the *j*-th risk factor, respectively.

We evaluated the performance of the model using 10-fold cross-validation. K-fold cross-validation was used to divide the data into K-folds of the same size, fit the prediction model with K − 1 folds as the training set, and measure the predictive performance of the fitted model with the remaining folds as the test set. Every fold is used as a test set once, and in this study, the predictive performance was measured using the average area under the receiver operating characteristic curve (AUC) [22].

## 3. Results

Table 1 presents the baseline patient characteristics. The number of 1000 person-years of follow-up was 49.16, with a median follow-up duration of 7.42 years. The mean age and BMI were 69.13 years and 23.92 kg/m^2^, respectively. The percentage of current smokers and heavy drinkers was 21.0% and 21.5%, respectively. The proportions of individuals who were engaged in low, moderate, and high physical activity were 71.9%, 5.2%, and 22.9%, respectively. Patients with multiple comorbid diseases (CCI 2 and ≥3) comprised 6.8% and 4.9% of the study population, respectively.

Table 2 summarizes the survival rates of prostate cancer patients after the initial diagnosis. The mean survival probabilities 1, 3, and 5 years after prostate cancer diagnosis were 0.955, 0.882, and 0.820, respectively.

The β-coefficients in the survival model function (1) and the hazard ratios in the prostate cancer survival models are presented in Table 3. Age, fasting glucose and GGT levels, current smoking, and CCI score were positively associated with mortality, whereas BMI, ALT level, total cholesterol level, alcohol intake, physical activity, and household income were inversely associated with mortality. Prediction models for the 5-year survival probability were constructed from the mean values in Table 1, the 5-year survival rate in Table 2, and the β-coefficients in Table 3. The risk prediction formulae for the 5-year survival of prostate cancer patients are as follows:

(1) Survival model function for men (SMFM)=0.0777×(age−69.13)−0.0477×(BMI−23.92)+0.0009×(SBP−127.89)+0.0023×(fasting glucose concentration−105.09)−0.0020×(ALT concentration−25.09)+0.0022×(GGT concentration−44.68)−0.0023×(total cholesterol concentration−186.67)−0.0082×(former smoker−0.352)+0.4020×(current smoker−0.210)−0.1563×(moderate drinker−0.268)−0.1189×(heavy drinker−0.215)−0.2592×(moderate exerciser−0.052)−0.2165×(heavy exerciser−0.229)−0.0505×(low income−0.161)−0.1126×(mid−low−0.202)−0.1753×(mid−high income−0.240)−0.2905×(high income−0.219)+0.1556×(CCI1−0.212)+0.5532×(CCI2−0.068)+1.0092×(CCI3−0.049)

(2) SMFM1 = exp (SMFM)

(3) 5-year survival probability = 1 − 0.820^SMFM1^

**Figure 2:**
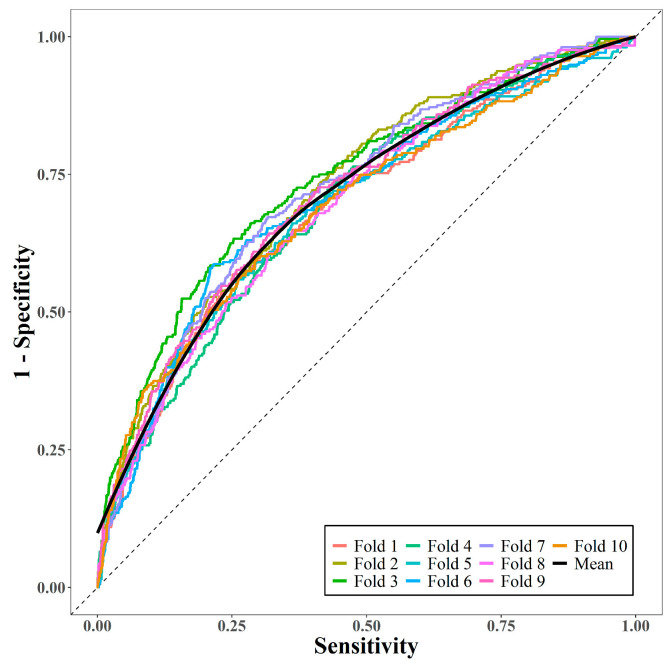
Area under receiver operating characteristic curves via 10-fold cross-validation.

The performance of the prediction model was measured via 10-fold cross-validation. Figure 2 displays the 10 receiver operating characteristic curves and the mean curve. The mean AUC was 0.71, suggesting that the model had moderately good predictive performance.

## 4. Discussion

In this study, a 5-year survival probability model for patients newly diagnosed with prostate cancer was developed using a nationwide insurance claims database. This model had good predictive performance based on the national health check-up data available in the primary care setting.

The age-standardized incidence rate of prostate cancer in Western and African countries is higher than that in Asian countries (62.1 in Europe, 73.7 in North America, 26.6 in Africa, and 11.5 in Asia per 100,000 individuals) [23]. Prostate cancer is the most common cancer in Japan, the United States (except skin cancer), and the United Kingdom, but ranks fourth in Korea [24]. Although the incidence rate in Korea is lower than that in other countries, it has steadily increased [4]. As many Koreans adopt westernized lifestyles and foods and grow older, the incidence will increase. The American Urology Association recommends PSA-based prostate cancer screening in combination with shared decision making for early detection [25]. However, the NHIS does not provide prostate cancer screening, as the National Cancer Screening Programs contain five types of cancer (stomach, liver, colorectum, breast, and uterine cervix) [5]. PSA-based prostate cancer screening can significantly increase the number of undiagnosed prostate cancer patients. If a PSA-based prostate cancer screening program is included in the National Cancer Screening Programs, the incidence of prostate cancer will increase significantly.

The recent 5-year relative survival rate for prostate cancer is 96.0% in Korea [4]. For these reasons, prostate cancer survivors will considerably increase in the near future.

Prostate cancer patients are more likely to die from cardiac, cerebrovascular, and chronic obstructive pulmonary diseases than from prostate cancer [26,27]. This likelihood is higher in patients with early-stage prostate cancer or in those who have survived a long time after diagnosis. Predicting the survival probability of prostate cancer patients is important for planning long-term care. If prostate cancer patients are expected to survive for a long time, their primary doctors should focus on preventing cardio-cerebrovascular diseases and second primary cancers as well as relieving their symptoms.

Although the 5-year relative survival rate among Korean prostate cancer patients diagnosed in 2017–2021 reached 96.0%, the survival rates 1, 3, and 5 years after the initial diagnosis were 95.5%, 88.2%, and 82.0%, respectively, in this cohort. This survival rate is comparable to that reported in another Korean study by Park et al. (2021) [28]. One reason for the possible underestimation in the present study is that patients diagnosed with prostate cancer between 2005 and 2016 were included. Other Korean studies reported a prognostic model [28,29]. Woo et al. developed a 5-year survival probability model for stomach cancer patients who underwent a gastrectomy [30]. This model is strong due to the inclusion of histopathological data, information about lymph node involvement, and surgical details; however, the reliance on detailed medical information makes it difficult for patients and primary care doctors to apply this model. Park et al. (2021) calculated the 5-year conditional relative survival and demonstrated competing mortality among patients with prostate cancer using a large cohort of >81,000 patients [28]. However, it is inconvenient to use this model in primary clinics because a numerical prognostic model was not developed [28]. Koo et al. (2020) developed an individualized survival prognostic model for patients newly diagnosed with prostate cancer [29]. The model was developed based on an artificial neural network model and included pathological stage, Gleason score, PSA levels, and anti-cancer treatment details such as surgery, radiation, androgen deprivation therapy, and performance scales [29]. While this model may more accurately predict survival outcomes compared to our model, it does not account for laboratory results, economic status, blood pressure, and lifestyle behaviors. In contrast, our model included other variables such as age, BMI, laboratory results, lifestyle behaviors, and economic status. These variables are easily available to nonmedical personnel or primary care doctors. In addition, these are commonly associated with cancer incidence, cardiovascular events, and mortality. Thus, patients with prostate cancer who are not medical personnel, their families, or primary care doctors are able to predict the 5-year survival probability, even without access to pathological and anti-cancer treatment details.

This study had several strengths. This retrospective cohort study included all patients newly diagnosed with prostate cancer between 2005 and 2016 based on nationwide insurance claims data. The Korea National Health Insurance Corporation (NHIC) is a non-profit organization and the single insurer that operates the NHIS to which all citizens must subscribe. The NHIC collects information from the NHIS and saves it in the NHID. The NHID contains nationwide claims and health check-up results, including anthropometric data and personal and familial medical histories. All data used in this study were obtained from the NHID. Thus, the study population is representative of Korean men with prostate cancer. Various confounders that may influence survival but are useful in primary care settings were included. Economic status, a major determinant of medical service use and accessibility, was considered when a patient’s survival probability was determined. Economic status was considered in our formula. Economic deprivation is associated with a higher cancer incidence and mortality [31,32,33]. Comorbidities, which were calculated as the CCI, were also reflected in the risk prediction formula, as the CCI is a measure of comorbid conditions related to patient death or medical resource utilization [19]. In addition, anthropometric data, laboratory results, and lifestyle behaviors were included in the formulae. Thus, this prognostic model for predicting the 5-year survival probability offsets the effects of unexpected confounders on the survival probability of prostate cancer patients.

However, limitations of this study should be considered when interpreting the results. First, histopathology, stage of prostate cancer, and treatment details, such as surgery, chemotherapy, hormonal therapy, and radiation therapy, were not available in the NHID. Although the inclusion of this information would allow for a more accurate prognosis, it would be difficult to use in primary care settings. Second, the survival rate relative to the time of cancer diagnosis was not exactly reflected because of the long inclusion period (2005–2016) and time-dependent improvement in the 5-year relative survival rate of prostate cancer (81.0% in 2001–2005 and 96.0% in 2016–2020) [4]. Third, no validation cohort was analyzed because the population size of prostate cancer patients was small. However, to mitigate the disadvantage of the lack of a validation cohort, we used 10-fold cross-validation to fit the model. Fourth, there may be imperfections in predictive modeling-based studies due to inaccurate variable measurements, insufficient clinical information, such as psychiatric problems, and a lack of supporting systems from residential areas and families. Continuous research using various population cohorts is needed to identify the most important variables related to death and to improve the accuracy of predictive models. Fifth, selection or healthy user bias should be considered. A combination of special codes for malignant neoplasms (V193 and V194) and prostate cancer based on the ICD-10 (C61) was used to minimize false positives in cancer diagnosis. However, patients with prostate cancer who underwent a national health check-up within 1 year before enrollment were included. The inclusion and exclusion criteria may have introduced bias.

## 5. Conclusions

A 5-year survival probability model for Korean men with prostate cancer was developed based on real-world data. This model may be useful for predicting the 5-year survival probability of prostate cancer patients in a primary care setting without the knowledge of the cancer stage, histological results, or treatment details.

## Figures and Tables

**Table 1:** Participants’ characteristics at baseline.

Category	Male
Number	14,228
Age, years	69.13 ± 6.97
Body mass index, kg/m^2^	23.92 ± 2.85
Systolic blood pressure, mmHg	127.89 ± 15.43
Fasting glucose, mg/dL	127.89 ± 27.39
Alanine aminotransferase, IU/L	25.09 ± 22.49
Gamma-glutamyl transferase, IU/L	44.68 ± 63.19
Total cholesterol, mg/dL	186.67 ± 39.11
Smoking status, n (%)	
Never smokers	6236 (43.8%)
Former smokers	5000 (35.2%)
Current smokers	2985 (21.0%)
Alcohol consumption, n (%)	
Rare	7363 (51.8%)
Moderate	3808 (26.8%)
Heavy	3057 (21.5%)
Physical activity, n (%)	
Low	10,229 (71.9%)
Moderate	741 (5.2%)
High	3258 (22.9%)
Household income, n (%)	
0th–20th	2542 (17.9%)
21st–40th	2294 (16.1%)
41st–60th	2870 (20.2%)
61st–80th	3412 (24.0%)
81st–100th	3110 (21.9%)
Charlson Comorbidity Index, n (%)	
0	9538 (67.0%)
1	3020 (21.2%)
2	966 (6.8%)
≥3	704 (4.9%)

Values are means ± standard deviations for continuous variables or number (%) for categorical variables.

**Table 2:** Survival rates among patients with prostate cancer.

Time since diagnosis	1 year	3 years	5 years
Number at risk	13,585	12,521	11,436
Number of deaths	634	1050	880
Survival rate (95% CIs *)	0.955 (0.952−0.959)	0.882 (0.876−0.887)	0.820 (0.813−0.826)

* Cis: confidence intervals.

**Table 3:** Hazard ratios and 95% confidence intervals for death among patients with prostate cancer.

Category	Coefficient	HR (95% CIs)
Age, years	0.0777	1.081 (1.075–1.086)
Body mass index, kg/m^2^	−0.0477	0.953 (0.944–0.963)
Systolic blood pressure, mmHg	0.0009	1.001 (0.999–1.003)
Fasting glucose, mg/dL	0.0023	1.002 (1.001–1.003)
Alanine aminotransferase, IU/L	−0.0020	0.998 (0.996–1.000)
Gamma-glutamyl transferase, IU/L	0.0022	1.002 (1.002–1.003)
Total cholesterol, mg/dL	−0.0023	0.998 (0.997–0.998)
Smoking status, n (%)		
Never smokers	0.000	Reference
Former smokers	−0.0082	0.992 (0.930–1.058)
Current smokers	0.4020	1.495 (1.394–1.603)
Alcohol consumption, n (%)		
Rare	0.000	Reference
Moderate	−0.1563	0.855 (0.798–0.916)
Heavy	−0.1189	0.888 (0.826–0.955)
Physical activity, n (%)		
Low	0.000	Reference
Moderate	−0.2592	0.772 (0.681–0.875)
High	−0.2165	0.805 (0.751–0.864)
Household income, n (%)		
0th–20th	0.000	Reference
21st–40th	−0.0505	0.951 (0.868–1.041)
41st–60th	−0.1126	0.894 (0.819–0.974)
61st–80th	−0.1753	0.839 (0.772–0.912)
81st–100th	−0.2905	0.748 (0.685–0.817)
Charlson Comorbidity Index, n (%)		
0	0.000	Reference
1	0.1556	1.168 (1.091–1.251)
2	0.5532	1.202 (1.582–1.911)
≥3	1.0092	2.743 (2.488–3.024)

Abbreviations: CI, confidence interval; HR, hazard ratio.

## Data Availability

The data supporting this article are accessible from the National Health Insurance Service’s Open Data Portal.
